# Nomograms of Combining MRI Multisequences Radiomics and Clinical Factors for Differentiating High-Grade From Low-Grade Serous Ovarian Carcinoma

**DOI:** 10.3389/fonc.2022.816982

**Published:** 2022-06-07

**Authors:** Cuiping Li, Hongfei Wang, Yulan Chen, Chao Zhu, Yankun Gao, Xia Wang, Jiangning Dong, Xingwang Wu

**Affiliations:** ^1^ Department of Radiology, The First Affiliated Hospital of Anhui Medical University, Hefei, China; ^2^ Department of Radiology, The First Affiliated Hospital of the University of Science and Technology of China, Anhui Provincial Cancer Hospital, Hefei, China; ^3^ Department of Radiotherapy, The First Affiliated Hospital, University of Science and Technology of China, Anhui Provincial Cancer Hospital, Hefei, China

**Keywords:** serous ovarian carcinoma, radiomics, nomogram, magnetic resonance imaging, subtype

## Abstract

**Objective:**

To compare the performance of clinical factors, FS-T2WI, DWI, T1WI+C based radiomics and a combined clinic-radiomics model in predicting the type of serous ovarian carcinomas (SOCs).

**Methods:**

In this retrospective analysis, 138 SOC patients were confirmed by histology. Significant clinical factors (*P* < 0.05, and with the area under the curve (AUC) > 0.7) was retained to establish a clinical model. The radiomics model included FS-T2WI, DWI, and T1WI+C, and also, a multisequence model was established. A total of 1,316 radiomics features of each sequence were extracted; the univariate and multivariate logistic regressions, cross-validations were performed to reduce valueless features and then radiomics signatures were developed. Nomogram models using clinical factors, combined with radiomics features, were developed in the training cohort. The predictive performance was validated by receiver operating characteristic curve (ROC) analysis and decision curve analysis (DCA). A stratified analysis was conducted to compare the differences between the combined radiomics model and the clinical model in identifying low- and high-grade SOC.

**Results:**

The AUC of the clinical model and multisequence radiomics model in the training and validation cohorts was 0.90 and 0.89, 0.91 and 0.86, respectively. By incorporating clinical factors and multi-radiomics signature, the AUC of the radiomic-clinical nomogram in the training and validation cohorts was 0.98 and 0.95. The model comparison results show that the AUC of the combined model is higher than that of the uncombined models (*P*= 0.05, 0.002).

**Conclusion:**

The nomogram models of clinical factors combined with MRI multisequence radiomics signatures can help identifying low- and high-grade SOCs and a provide a more comprehensive, effective method to evaluate preoperative risk stratification for SOCs.

## Introduction

Ovarian cancer (OC) is the deadliest type of gynecological malignancy worldwide (1), and SOC is the most common histological type, accounting for approximately 70% of cases (2). High-grade serous ovarian carcinomas (HGSOCs) are the largest category of OC, comprising 90% of the total, and have high recurrence and mortality rates (3, 4). In contrast, LGSOCs are much less common, accounting for less than 10% of the remaining cancers and have a strong association with borderline tumors (2). Some previous studies have shown that apparent diffusion coefficient (ADC) histogram parameters based on whole solid tumor volume could be helpful for differentiating between HGSOC and LGSOC (2), single or multisequence MRI-based texture features of the whole tumor also might assist in characterizing the differences between borderline tumors and LGSOCs (5), and based on whole solid-tumor histogram analysis, could effectively differentiate benign OCs from malignant OCs (6). HGSOCs and LGSOCs are two separate tumors with distinct genetic risk factors, epidemiological differences, biological behaviors, different spreading patterns, response to chemotherapy, and prognosis (3, 7). Moreover, accurate preoperative diagnosis of subtypes will be helpful for achieving a more effective subtype specific treatment. However, no combination of radiomics and clinical models has been used to distinguish HGSOCs from LGSOCs. Based on the current study progress and the necessity of solving clinical problems, the purpose of the present study was to compare the performance of clinical factors, fat-suppressed T2-wighted imaging (FS-T2WI), diffusion-weighted imaging (DWI), contrast-enhanced FS T1WI (T1WI+C) radiomics, and a combined multiple features model in predicting the subtype of SOCs.

## Materials and Methods

### Patients

The present retrospective study was approved by the institutional review board and the need for an informed patient consent was waived. Between December 2014 and August 2019, a total of 226 patients diagnosed clinically with OCs were identified. In order to eliminate the effects of the MRI parameters and OC subtype on the results, the investigators only included patients with LG- and HGSOCs scanned on the same MRI platform with a unified imaging protocol (n=185). Then, investigators excluded the following patients: (1) MRI contraindications or MRI quality that cannot meet the diagnostic requirements, (2) patients treated with neoadjuvant chemotherapy, and (3) a tumor with few solid components. Finally, 138 patients with ovarian cancers (104 HGSOCs and 34 LGSOCs) confirmed by surgery and histology were included. The mean age was 54.83 ± 11.04 years. The tumors were staged according to the 2014 International Federation of Gynecology and Obstetrics (FIGO) staging system. The process of patient selection is illustrated in **Figure S1**.

### MRI Protocol

MRI examination was performed using a unit system (GE Signa HDXT 3.0T MRI scanner, GE Healthcare, USA) equipped with an 8-channel phased-array abdominal coil. Excluding contraindications, all patients received an intramuscular injection of 15 mg hyoscine butylbromide at 30 minutes before the MRI scan to prevent gastrointestinal motility. The bladder was kept approximately half-filled, in order to improve lesion visibility without changing the anatomy. Patients were placed in the supine position and were breathing freely during the acquisition.

The routine pelvic MRI protocol consisted of the following sequences: axial T1-weighted imaging (T1WI), axial/sagittal T2-weighted imaging (T2WI), axial FS T2WI, DWI (*b* value = 0, 1,000 s/mm^2^), and multiple phases of contrast-enhanced (LAVA-FLEX) MRI. When scanning the axial images, the transverse plane was perpendicular to the long axis of the uterine body and for the sagittal images, the longitudinal plane was parallel to the main body of the uterus. T1WI+C sequence was acquired at the arterial, venous, and delayed phases of contrast medium enhancement in axial planes, which were acquired at 25, 60, and 120 s after the intravenous injection of 0.1 mmol/kg gadodiamide (Omniscan, GE Healthcare) using an Ulrich power injector. The scanning sequences and parameters are shown in **Table S1**.

### MRI Images Analysis

**Figure 1:**
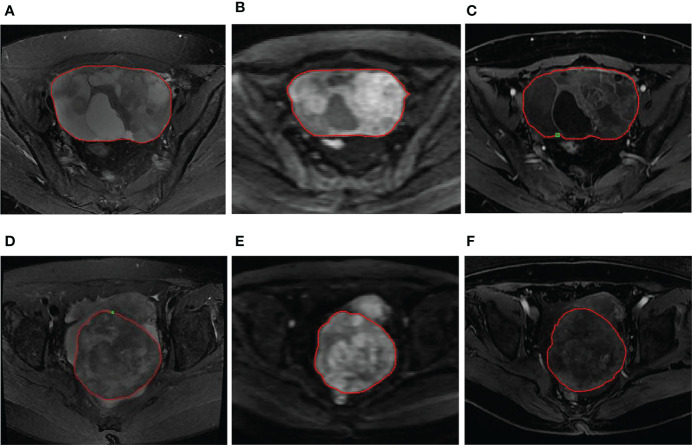
**(A–C)** A 56-year-old woman with LGSOC in the right ovary. **(A)** Axial FS-T2WI shows a mixed cystic-solid mass. **(B)** Axial DWI (b=1000 s/mm^2^) shows high signal of the solid component, indicating limited diffusion. **(C)** Axial T1WI+C image shows a mild enhancement in the solid component and septation. The red ROI was manually drawn along the margin of the whole tumor. **(D–F)** A 63-year-old woman with HGSOC in the right ovary. **(D)** Axial FS-T2WI shows a mass with mixed signals dominated by solid components. **(E)** Axial DWI (b=1000 s/mm^2^) shows high signal of the tumor, indicating limited diffusion. **(F)** Axial T1WI+C image shows a heterogeneous mild enhancement in the tumor. The red ROI was manually drawn along the margin of the whole tumor.

Two radiologists with more than 10 years of experience in gynecological imaging analyzed the images without knowing the pathological results of these patients and reached a consensus (**Figure 1**). Using the GE ADW 4.6 post-processing workstation, the DWI images of the tumor layer with b = 1000 s/mm^2^ were analyzed and the ADC values were calculated. The measurement was repeated three times and the average value was obtained. When sketching for the region of interest (ROI), the T2WI and T1WI+C images were referenced to determine the tumor boundary and the mucus, necrosis, cystic change, and bleeding areas were avoided.

### MRI Image Segmentation and Radiomics Feature Extraction

Manual segmentation was performed based on FS-T2WI, DWI, and T1WI+C sequences by using the ITK-SNAP software (version 3.8.0, www.itksnap.org). The region of interest (ROI) of each ovarian tumor was manually contoured along the boundary of the tumor and the VOI was constructed by ROI interpolation for each slice. The interobserver reproducibility was initially analyzed using 30 randomly chosen images for the VOI by the 2 radiologists mentioned above. Intraclass correlation coefficients (ICCs) were used to evaluate the interobserver agreement in the measurement of radiomics features (ICC>0.75 was indicative of almost perfect agreement).

**Figure 2:**
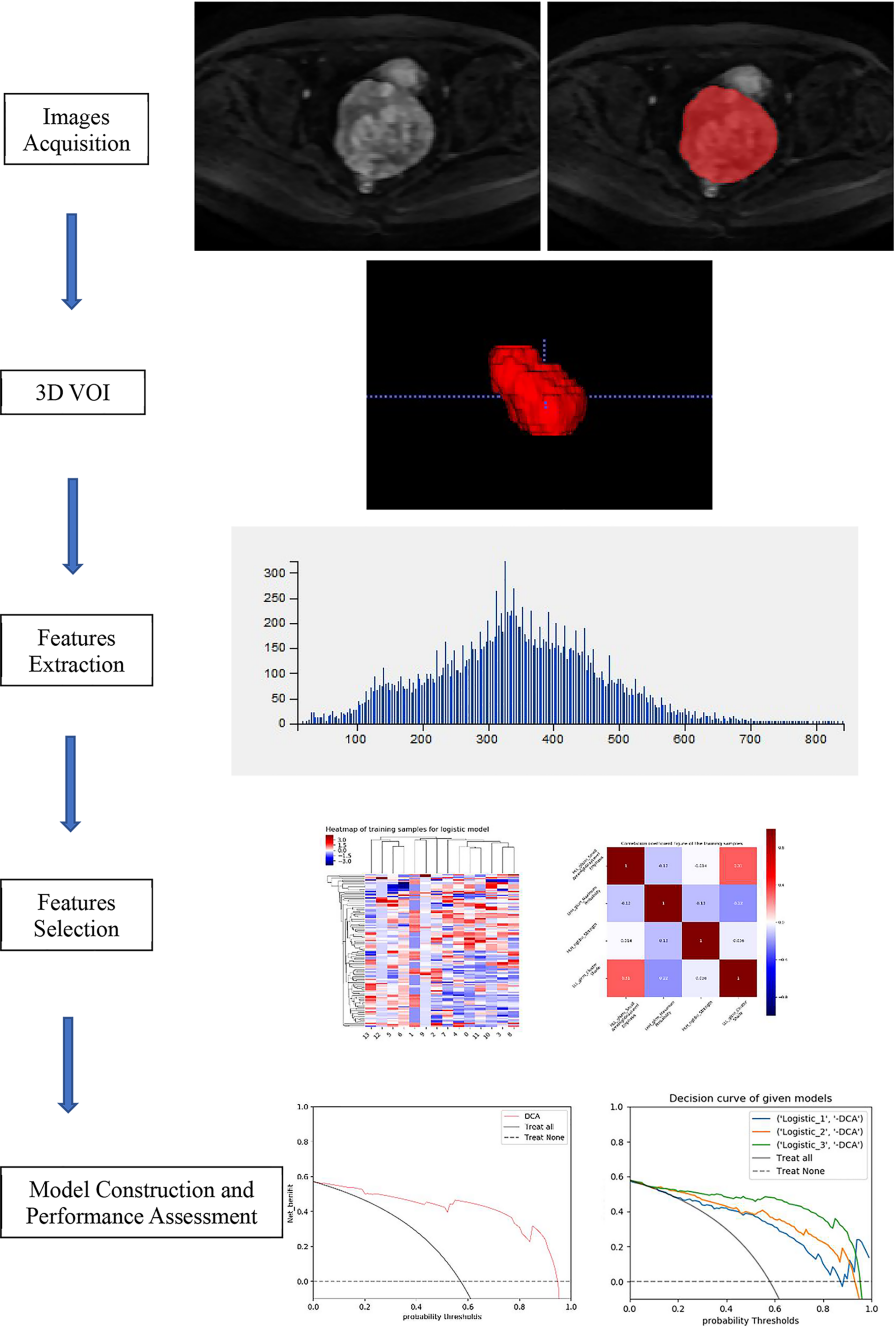
The detailed description of the radiomics images preprocessing.

To reduce the discrepancies between imaging parameters, several preprocessing steps of the MR images were applied before the process of radiomics feature extraction. All images were resampled to a voxel size of 1×1×1 mm^3^ using B-Spline interpolation. Each MRI scan was normalized in order to get a standard normal distribution of image intensities. Radiomics features were extracted from 3 types of multisequence MR images (FS-T2WI, DWI, and T1WI+C) for each lesion using PyRadiomics software http://pyradiomics.readthedocs.io/en/latest/index.html, which could automatically obtain the histogram parameters for whole solid tumor VOI. Seven classes of 1,316 radiomics features were extracted: shape features (2D, 3D), first-order features, gray-level cooccurrence matrix (GLCM) features, gray-level run length matrix (GLRLM) features, gray-level size zone matrix (GLSZM) features, neighborhood gray-tone difference matrix (NGTDM) features, and gray-level dependence matrix (GLDM) features (**Table S2**). The detailed description of the radiomics images preprocessing is shown in **Figure 2**.

### Data Preprocessing

The dataset was randomly assigned in a 7:3 ratio to either the training cohort or validation cohort. All cases in the training cohort were used to train the predictive model, while cases in the validation cohorts were used to independently evaluate the model’s performance.

Before analyses, variables with zero variance were excluded from analyses. Then, the missing values and outlier values were replaced by the median. Finally, the data were standardized by the standardization.

### Feature Selection and Classifier Modeling

**Figure 3:**
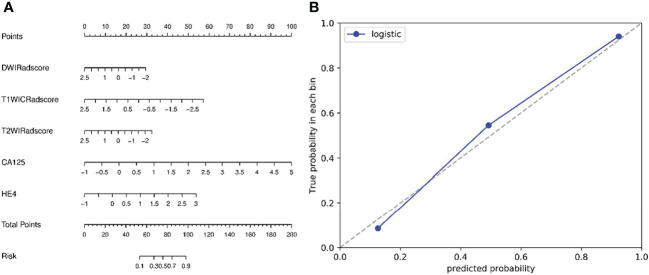
**(A)** Nomogram based on radiomics signatures and clinical factors. In the nomogram, a vertical line was made according to each parameter to determine the corresponding value of points. The total points were the sum of the three points above. Then, a vertical line was made according to the value of the total points to determine the probability type of SOCs. **(B)** Calibration curves of the nomogram model (clinical factors + multisequence radiomics signatures) in validation cohort. The 45° dotted line represents the ideal prediction, while the blue line represents the prediction performance of the nomogram. The closer the blue line is to the dotted line, the better the performance of the nomogram.

Firstly, features with ICCs >0.75 were retained. Secondly, feature selection was performed by using univariate logistic analysis (Correlation_xx), multivariate logistic analysis, and gradient boosting decision tree (GBDT) with stepwise selection method. The rad-score value was calculated by the sum of (radiomics signature × coefficient) + intercept. Finally, logistic-based rad-score model and nomogram was built based on the established optimal feature subsets of the training cohort (**Figure 3**).

**Figure 4:**
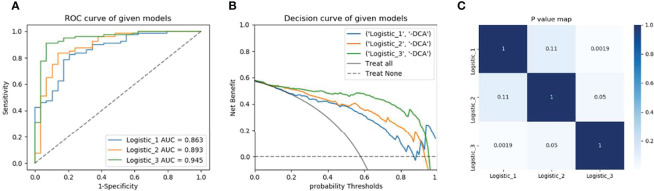
Model’s performance assessment and comparison. **(A)** Receiver operating characteristic curve analysis in the validation cohorts. **(B)** Decision curve analysis of radiomics signature, clinical model, and nomogram respectively. **(C)** Delong test for the given models. Model 1is based on MRI multisequence radiomics Model 2 is based on clinical factors and Model 3 is a combination of clinical and multi-radiomics.

Receiver operating characteristic (ROC) curve was performed to determine the performance of the machine learning model; accuracy, sensitivity, specificity and area under curve (AUC) was calculated. The differences for radiomics models were compared using the DeLong method (**Figure 4**).

### Statistical Analysis

A commercial software (SPSS 22.0, IBM Corporation, Armonk NY, USA) was used for the statistical analysis. We tested whether the numerical variables were normally distributed by using a one-sample Kolmogorov-Smirnov test. The data that had a normal distribution were expressed as mean ± standard deviation (SD), while nonnormally distributed data were expressed in median (interquartile range (IQR), 25th and 75th percentile). Independent sample *t*-test was used to conform to the normal distribution, while Mann-Whitney *U*-test in the nonparametric rank sum test was used to conform to the nonnormal distribution. The Chi-square test was used for unordered categorical variables. *P*<0.05 was considered as a statistically significant.

## Results

### Clinical Factors Analysis

**Table 1** reports the comparisons of clinical factors between HGSOCs and LGSOCs. The preoperative carbohydrate antigen 125 (CA125) and human epididymis protein 4 (HE4) indicators in HGSOCs are significantly higher than that of LGSOCs (801.900 (381.100, 2066.750) VS 161.600 (80.622, 422.550) *P*<0.001, 429.550 (213.875, 922.850) VS 109.800 (70.795, 185.825) *P*<0.001, respectively). The ADC value in HGSOCs is 0.865 (0.743, 9.955), which is significantly lower than that of LGSOCs (0.980 (0.817, 1.110), *P*<0.001). No significant difference is found between HGSOCs and LGSOCs in age, peritoneal metastasis (PM), lymph node metastasis (LNM), and location. Based on the variables mentioned above, a clinical model was established, the AUC was 0.89 (95% confidence interval [CI] 0.78–0.96).

**Table 1:** Demographic and clinicopathologic characteristics of patients with serous ovarian carcinomas.

Variables	LGSOC (n=34)	HGSOC (n=104)	*P* value
Ages, median (IQR)	52 (45, 62)	55 (50, 64)	0.298^1^
Overall FIGO stage, N (%)			
IA	0 (0)	2 (1.9%)	
IB	1 (2.9%)	2 (1.9%)	
IC	6 (17.6%)	10 (9.6%)	
IIA	1 (2.9%)	0 (0)	
IIB	2 (5.9%)	5 (4.8%)	
IIIA	3 (8.8%)	6 (5.8%)	
IIIB	2 (5.9%)	6 (5.8%)	
IIIC	9 (26.5%)	52 (50.0%)	
IVA	4 (11.8%)	6 (5.8%)	
IVB	6 (17.6%)	15 (14.4%)	
ADC value, median (IQR)	0.980 (0.817, 1.110)	0.865 (0.743, 9.955)	<0.001^1^
CA125, median (IQR)	161.600 (80.622, 422.550)	801.900 (381.100, 2066.750)	<0.001^1^
HE4, median (IQR)	109.800 (70.795, 185.825)	429.550 (213.875, 922.850)	<0.001^1^
Location, N (%)			
Bilateral	20 (58.8%)	46 (44.2%)	0.139^2^
Unilateral	14 (41.2%)	58 (55.8%)	
LNM, N (%)			
–	19 (55.9%)	39 (37.5%)	0.059^2^
+	15 (44.1%)	65 (62.5%)	
PM, N (%)			
–	14 (41.2%)	25 (24.0%)	0.054^2^
+	20 (58.8%)	79 (76.0%)	

LGSOC, low-grade serous ovarian carcinoma; HGSOC, high-grade serous ovarian carcinoma; IQR, interquartile range; FIGO, international federation of gynecology and obstetrics; ADC, apparent diffusion coefficient; CA125, carbohydrate antigen 125; HE4, human epididymis protein 4; LNM, lymph node metastasis; PM, peritoneal metastasis. ^1^Mann-Whitney U test, ^2^Chi-square test.

### Radiomics Models and Model Comparisons

In total, 1,316 radiomics features were extracted from each VOI of the three sequences. The ICC values range from 0.771 to 0.988 and show great interobserver agreement. Four, eight, and six features were extracted from DWI, T1WI+C, and FS-T2WI sequences, respectively (**Table S3**). Based on the above features, the radiomics models of single sequence and multisequence combinations are established, respectively. The AUC of the combined radiomics model was 0.86 (95% CI 0.81–0.97), ACC 0.79. The combination of signatures from the multisequence resulted in a better predictive model than that from a single sequence (**Table 2**).

**Table 2:** Performance evaluation of the models.

	Training cohort						Validation cohort					
	AUC	ACC	SEN	SPE	PPV	NPV	AUC	ACC	SEN	SPE	PPV	NPV
Clinical model	0.90 (0.84, 0.95)	0.83	0.78	0.90	0.91	0.75	0.89 (0.78, 0.96)	0.85	0.91	0.77	0.84	0.87
DWI Radiomics	0.84 (0.73, 0.92)	0.78	0.71	0.85	0.83	0.75	0.83 (0.76, 0.89)	0.75	0.70	0.79	0.77	0.73
DCE Radiomics	0.89 (0.81, 0.95)	0.80	0.64	0.96	0.95	0.73	0.86 (0.80, 0.91)	0.78	0.65	0.92	0.89	0.72
FS-T2WI Radiomics	0.87 (0.81, 0.93)	0.83	0.89	0.77	0.79	0.88	0.83 (0.73, 0.92)	0.74	0.72	0.72	0.87	0.67
Multi- Radiomics	0.91 (0.83, 0.97)	0.87	0.73	0.87	0.87	0.72	0.86 (0.81, 0.97)	0.78	0.83	0.72	0.80	0.76
Nomogram	0.98 (0.93, 0.99)	0.90	0.91	0.90	0.92	0.88	0.95 (0.90, 0.98)	0.87	0.91	0.83	0.90	0.77

AUC, area under the curve; ACC, accuracy; SEN, sensitivity; SPE, specificity; PPV, positive predictive value; NPV, negative predictive value.

Then, the Rad-score was calculated for the radiomics features of individual sequences, then new combination model and Nomogram were established with combining clinical factors. The diagnostic efficacy of the combined model was higher than that of the clinical model or radiomics model (AUC = 0.95 (95% CI 0.90-0.98), ACC 0.87. Finally, the DeLong test results of model comparison show that the performance of the combined model is better than that of the uncombined models (*P*= 0.002, 0.05).

## Discussion

Tumor heterogeneity is one of the most important characteristics of malignant tumors (8, 9). It refers to the changes in molecular biology or genes of its daughter cells after multiple division and proliferation in the growth process of the tumor, resulting in differences in tumor growth rate, aggressive behaviors, response to treatment, and other aspects. In general, HGSOCs generally exhibit more aggressive behavior than LGSOCs (3). As a result, patients with HGSOCs have a poor prognosis, with a five-year survival rate of less than 40% (10). The optimal treatments for patients with HGSOCs include comprehensive staging surgery, cytoreductive surgery, and platinum-based chemotherapy (11). Most HGSOC patients, in contrast to LGSOCs, show an initially higher chemosensitivity (3, 12). LGSOCs typically occur in younger women with slow-growing behaviors (3). The treatment strategy of LGSOCs mainly rely on optimal surgical cytoreduction for best long-term survival (2). For patients in advanced stage, the formulation of surgical plan is also very important, not only to maximize the removal of tumor tissue, but also to take into account the postoperative quality of life of patients. Thus, accurate preoperative subtype diagnosis will be necessary for achieving a more effective subtype-specific treatment.

Conventional magnetic resonance imaging (cMRI) plays a significant role in the diagnosis of pelvic diseases due to its high soft-tissue resolution (13, 14). It can clearly reveal the lesion characteristics (cystic, bleeding, edema, fibrosis, and so on), the relationship between the tumor and surrounding tissues, and the status of lymph node disease (metastasis or inflammation). Some previous studies have shown that the qualitative diagnosis of EOCs by MRI is obviously superior to other imaging methods (10), but HGSOC and LGSOC images have many overlapping signs on cMRI (T1WI, T2WI, T1WI+C) sequences, and as a result, it is difficult for experienced radiologists to make a definite diagnosis. In addition, cMRI relies on the experience of radiologists and to some extent lacks objectivity (3). Here, especially the novel technique of DWI, has been widely used to investigate the heterogeneity of tumors in many clinical studies (1, 15–17). DWI can reflect the microbiological state of tissues noninvasively, according to the Brownian motion of water molecule, and indirectly reflect the biological changes in tissues (17–19). Radiomics analysis is a postprocessing method for extracting information by quantifying the spatial distribution of pixels or voxels with different gray intensities and counting the variables, that is, calculating and extracting texture features based on the texture matrix of images (10, 20, 21). However, some previous studies only analyzed simple parameter values, used different ROIs, including round, single-slice, and whole solid tumor volume, for OC analysis (3, 16, 17, 21, 22). This approach inevitably leads to a loss of important heterogeneous information. Considering the large size and complexity of ovarian masses, histogram analysis based on whole solid tumor VOI could more accurately reflect the heterogeneity of OCs by quantifying complex parameter distributions and provide a more accurate information for clinical practice (3, 17).

Our present study developed a series of predictive models based on clinical factors combined with radiomics analysis based on FS-T2WI, DWI, and T1WI+C sequences to provide a practical clinical tool for risk stratification and individualized treatment of SOCs. In the present study, patients with HGSOCs have higher levels of serum CA125 (801.900 (381.100, 2066.750) VS 161.600 (80.622, 422.550), *P*<0.001) and HE4 (429.550 (213.875, 922.850) VS 109.800 (70.795, 185.825), *P*<0.001), these results are consistent with the findings reported in previous studies (1, 12, 23). Indicating that the faster and more aggressive the tumor, the higher the levels of CA125 and HE4 secreted by the body. In other studies, CA125 has been shown to contribute to the early detection of asymptomatic OCs patients, leading to early diagnosis and treatment and eventually improve the prognosis of patients (4, 24). Other studies have pointed that CA125 has an advantage in distinguishing OCs from healthy individuals, but HE4 is more sensitive in distinguishing OCs from benign pelvic masses. And CA125 levels were strongly correlated with clinical evolution during chemotherapy. Not all OCs express CA125 abnormally and HE4 may be a useful addition to the test (23, 25). In addition, the ADC values of HGSOC patients are lower than that of LGSOC patients (0.865 (0.743, 9.955) VS 0.980 (0.817, 1.110), *P*<0.001, AUC<0.7), indicating that HGSOCs have higher tumor cell density and vigorous growth, which is also related to the high invasiveness and high recurrence rate of HGSOCs (1, 2, 12). Too many studies have found similar results, which is why DWI is widely used in clinical practice. However, there was no significant difference in age between the two groups in our study, HGSOC patients are slightly older than LGSOC patients (55 (50, 64) VS 52 (45, 62), *P*=0.298), which is different from the known views (3, 17, 26). The possible reason is that the clinical onset of SOCs is insidious, LGSOCs are less invasive, have a longer course of disease, and the symptoms are severe when hospitalized (16). In the future, we should focus on ‘high alert’ symptoms: pelvic or abdominal pain, increase abdominal size or bloating and difficulty eating/feeling full (27).

Eighty percent of SOC patients already have metastatic disease at diagnosis, with poor prognosis despite surgery and chemotherapy (28). In the present study, there were no significant differences in tumor location, peritoneal, and lymph node metastasis between the two groups. The present study showed that HGSOC patients were more likely to be unilateral (58/104, 55.8%), while LGSOC patients were slightly more likely to be bilateral (20/34, 58.8%). Our findings are consistent with previous studies that show HGSOC arises from the epithelium of the fallopian tube, whereas LGSOC usually arises from the ovary and is often bilateral (2, 24). Both HGSOC and LGSOC patients are prone to peritoneal metastasis, and HGSOC is more likely to occur (79/104, 76.0% VS 20/34, 58.8%). This may be related to the fact that the two subtypes are often found and treated in an advanced stage (29, 30). Lymph node metastasis was more common in HGSOC patients (65/104, 62.5%) than in LGSOC patients (15/34, 44.1%). The reason may be related to the late stage of patients enrolled in the present study, the high invasiveness of HGSOCs, and the small number of patients in the LGSOCs group. If patients with SOCs can achieve early detection and carry out relevant research at the early stage of the disease, the prognosis should be better. The clinical model based on the above clinical factors were built in the training cohorts to predict the type of SOCs, and validated to have good accuracy, sensitivity, and specificity in the validation cohorts.

In the other part of our study, 4 DWI features, 8 T1WI+C features, and 6 FS-T2WI features were screened out from radiomics analysis, after dimensionality reduction by the univariate, and multivariate logistic regression method, respectively. These radiomics represent the heterogeneity of SOC tumors and reflect the size and spatial distribution of gray level. HGSOCs are more heterogeneous than LGSOCs, which associate with vigorous mass growth, high aggressiveness, and high recurrence. Previous studies have found similar findings, and a convincing explanation is the HGSOCs possess more tortuous vascular structure and heterogeneous cellular morphology, which contribute to intratumoral parenchyma heterogeneity (1, 12, 31). Single sequence and multiple sequences models were established by using the selected radiomics features. After model comparison and DeLong’s test, the results show that the multisequence combination model has better performance, higher accuracy and sensitivity (0.86 (0.81, 0.97), 0.78, 0.83), the model based on T1WI+C signatures has the highest specificity (0.92), and all *P* values were less than 0.05. It has been proved that features based on image texture high-throughput extraction can more objectively and effectively predict the diagnosis, pathological grade, histological classification, lymph node metastasis, and prognosis of different diseases (20, 22, 32, 33). Combined with clinical factors and radiomics features, the nomogram was further established. The results show that the combined model had higher accuracy and sensitivity (0.95 (0.90, 0.98), 0.87, 0.91), but was less specific than the T1WI+C model (0.83 VS 0.92), and the difference was statistically significant (*P* = 0.002, 0.05, DeLong’s test). The results of this study indicate that the nomogram of multisequence features combined with clinical factors has a high diagnostic performance in distinguishing HGSOCs from LGSOCs. Due to the silencing of OCs growth, the tumor was large when it was discovered, accompanied by obvious cystic degeneration, necrosis, hemorrhage, and other manifestations. The tumors performed with obvious heterogeneity and it was difficult to distinguish subtypes only by clinical factors and the differentiation of subtypes was closely related to clinical treatment strategies. Previous studies have mostly used conventional plain scanning of single T2WI sequence, which may lead to the loss of some important features. The combination of non-contrast-enhanced sequences, T1WI+C, and DWI of functional sequences can better delineate the entire tumor profile and perform more detailed imaging analysis of the entire tumor, thus obtaining higher diagnostic value.

There are several limitations in the present study. First of all, this study is a retrospective single-center study and the results may be influenced by sample selection. Therefore, prospective randomized trials with a larger sample, especially external validation, are warranted to validate the generalization capabilities of the prediction model. Secondly, the number of LGSOCs patients is relatively small, which is mainly related to the low morbidity. Finally, manual sketching of VOI was adopted in this study and errors were unavoidable. Further studies are needed to expand the sample size, gradually combine with automatic sketching technology instead of manual sketching VOI, and improve the robustness of the study.

## Conclusion

In summary, we developed and validated a nomogram model combined with MRI-based multisequence radiomics signatures and clinical factors for the individualized prediction of type in SOCs and showed a favorable prediction performance. The nomogram models provided us a more comprehensive, effective method to evaluate risk stratification for SOCs, and could further help clinicians to specify personalized treatment strategies to improve patients’ prognosis.

## Data Availability Statement

The raw data supporting the conclusions of this article will be made available by the authors, without undue reservation.

## Ethics Statement

The studies involving human participants were reviewed and approved by Anhui Provincial Hospital Ethics Committee. Written informed consent for participation was not required for this study in accordance with the national legislation and the institutional requirements.

## Author Contributions

XwW, JD, CL, XW, CZ, and YG: conception and design. CL, YC, CZ, YG, and HW: collection and arrangement of data. CL, CZ, YG, XwW, and JD: data analysis and manuscript writing. All authors contributed to the article and approved the submitted version.

## Funding

This work was supported by 2020 SKY Image Research Fund (NO. Z-2014-07-2003-11).

## Conflict of Interest

The authors declare that the research was conducted in the absence of any commercial or financial relationships that could be construed as a potential conflict of interest.

## Publisher’s Note

All claims expressed in this article are solely those of the authors and do not necessarily represent those of their affiliated organizations, or those of the publisher, the editors and the reviewers. Any product that may be evaluated in this article, or claim that may be made by its manufacturer, is not guaranteed or endorsed by the publisher.

## References

[1] Qian L Ren J Liu A Gao Y Hao F Zhao L. MR Imaging of Epithelial Ovarian Cancer: A Combined Model to Predict Histologic Subtypes. Eur Radiol (2020) 30:5815–25. doi: 10.1007/s00330-020-06993-5 32535738

[2] Kawaguchi M Kato H Hatano Y Tomita H Hara A Suzui N. MR Imaging Findings of Low-Grade Serous Carcinoma of the Ovary: Comparison With Serous Borderline Tumor. Jpn J Radiol (2020) 38:782–9. doi: 10.1007/s11604-020-00960-2 32246351

[3] Li HM Zhang R Gu WY Zhao SH Lu N Zhang GF. Whole Solid Tumour Volume Histogram Analysis of the Apparent Diffusion Coefficient for Differentiating High-Grade From Low-Grade Serous Ovarian Carcinoma: Correlation With Ki-67 Proliferation Status. Clin Radiol (2019) 74:918–25. doi: 10.1016/j.crad.2019.07.019 31471063

[4] Sopik V Iqbal J Rosen B Narod SA. Why Have Ovarian Cancer Mortality Rates Declined? Part I. Incidence Gynecol Oncol (2015) 138:741–9. doi: 10.1016/j.ygyno.2015.06.017 26080287

[5] Kurokawa R Nakai Y Gonoi W Mori H Tsuruga T Makise N. Differentiation Between Ovarian Metastasis From Colorectal Carcinoma and Primary Ovarian Carcinoma: Evaluation of Tumour Markers and “Mille-Feuille Sign” on Computed Tomography/Magnetic Resonance Imaging. Eur J Radiol (2020) 124:108823. doi: 10.1016/j.ejrad.2020.108823 31935596

[6] Turkoglu S Kayan M. Differentiation Between Benign and Malignant Ovarian Masses Using Multiparametric MRI. Diagn Interv Imaging (2020) 101:147–55. doi: 10.1016/j.diii.2020.01.006 31987805

[7] Delgado Bolton RC Aide N Colletti PM Ferrero A Paez D Skanjeti A. EANM Guideline on the Role of 2-[18F]FDG PET/CT in Diagnosis, Staging, Prognostic Value, Therapy Assessment and Restaging of Ovarian Cancer, Endorsed by the American College of Nuclear Medicine (ACNM), the Society of Nuclear Medicine and Molecular Imaging (SNMMI) and the International Atomic Energy Agency (IAEA). Eur J Nucl Med Mol Imaging (2021) 48:3286–302. doi: 10.1007/s00259-021-05450-9 34215923

[8] Song XL Ren JL Zhao D Wang L Ren H Niu J. Radiomics Derived From Dynamic Contrast-Enhanced MRI Pharmacokinetic Protocol Features: The Value of Precision Diagnosis Ovarian Neoplasms. Eur Radiol (2021) 31:368–78. doi: 10.1007/s00330-020-07112-0 32767049

[9] Torre LA Trabert B Desantis CE Miller KD Samimi G Runowicz CD. Ovarian Cancer Statistics, 2018. CA Cancer J Clin (2018) 68:284–96. doi: 10.3322/caac.21456 PMC6621554 29809280

[10] Li H Zhang R Li R Xia W Chen X Zhang J. Noninvasive Prediction of Residual Disease for Advanced High-Grade Serous Ovarian Carcinoma by MRI-Based Radiomic-Clinical Nomogram. Eur Radiol (2021) 31:7855–64. doi: 10.1007/s00330-021-07902-0 33864139

[11] Mulligan KM Glennon K Donohoe F O’brien Y Donnell BC Bartels HC. Multidisciplinary Surgical Approach to Increase Complete Cytoreduction Rates for Advanced Ovarian Cancer in a Tertiary Gynecologic Oncology Center. Ann Surg Oncol (2021) 28:4553–60. doi: 10.1245/s10434-020-09494-3 33423175

[12] Wang F Wang Y Zhou Y Liu C Xie L Zhou Z. Comparison Between Types I and II Epithelial Ovarian Cancer Using Histogram Analysis of Monoexponential, Biexponential, and Stretched-Exponential Diffusion Models. J Magn Reson Imaging (2017) 46:1797–809. doi: 10.1002/jmri.25722 28379611

[13] Rizzo S Del Grande M Manganaro L Papadia A Del Grande F. Imaging Before Cytoreductive Surgery in Advanced Ovarian Cancer Patients. Int J Gynecol Cancer (2020) 30:133–8. doi: 10.1136/ijgc-2019-000819 31754068

[14] Wei M Bo F Cao H Zhou W Shan W Bai G. Diagnostic Performance of Dynamic Contrast-Enhanced Magnetic Resonance Imaging for Malignant Ovarian Tumors: A Systematic Review and Meta-Analysis. Acta Radiol (2021) 62:966–78. doi: 10.1177/0284185120944916 32741199

[15] Li Y Jian J Pickhardt PJ Ma F Xia W Li H. MRI-Based Machine Learning for Differentiating Borderline From Malignant Epithelial Ovarian Tumors: A Multicenter Study. J Magn Reson Imaging (2020) 52:897–904. doi: 10.1002/jmri.27084 32045064

[16] Song XL Ren JL Yao TY Zhao D Niu J. Radiomics Based on Multisequence Magnetic Resonance Imaging for the Preoperative Prediction of Peritoneal Metastasis in Ovarian Cancer. Eur Radiol (2021) 11:8438–46. doi: 10.1007/s00330-021-08004-7 33948702

[17] Li HM Tang W Feng F Zhao SH Gu WY Zhang GF. Whole Solid Tumor Volume Histogram Parameters for Predicting the Recurrence in Patients With Epithelial Ovarian Carcinoma: A Feasibility Study on Quantitative DCE-MRI. Acta Radiol (2020) 61:1266–76. doi: 10.1177/0284185119898654 31955611

[18] Winfield JM Wakefield JC Dolling D Hall M Freeman S Brenton JD. Diffusion-Weighted MRI in Advanced Epithelial Ovarian Cancer: Apparent Diffusion Coefficient as a Response Marker. Radiology (2019) 293:374–83. doi: 10.1148/radiol.2019190545 31573402

[19] Ye Z. Editorial for “Histogram Analysis Comparison of Monoexponential, Advanced Diffusion- Weighted Imaging, and Dynamic Contrast-Enhanced MRI for Differentiating Borderline From Malignant Epithelial Ovarian Tumors”. J Magn Reson Imaging (2020) 52:269–70. doi: 10.1002/jmri.27120 32134534

[20] Li C Zheng M Zheng X Fang X Dong J Wang C. Predictive Ki-67 Proliferation Index of Cervical Squamous Cell Carcinoma Based on IVIM-DWI Combined With Texture Features. Contrast Media Mol Imaging (2021) 2021:8873065. doi: 10.1155/2021/8873065 33531882 PMC7826202

[21] Ye R Weng S Li Y Yan C Chen J Zhu Y. Texture Analysis of Three-Dimensional MRI Images May Differentiate Borderline and Malignant Epithelial Ovarian Tumors. Korean J Radiol (2021) 22:106–17. doi: 10.3348/kjr.2020.0121 PMC7772386 32932563

[22] Nougaret S Mccague C Tibermacine H Vargas HA Rizzo S Sala E. Radiomics and Radiogenomics in Ovarian Cancer: A Literature Review. Abdom Radiol (NY) (2021) 46:2308–22. doi: 10.1007/s00261-020-02820-z 33174120

[23] Yang WL Lu Z Guo J Fellman BM Ning J Lu KH. Human Epididymis Protein 4 Antigen-Autoantibody Complexes Complement Cancer Antigen 125 for Detecting Early-Stage Ovarian Cancer. Cancer (2020) 126:725–36. doi: 10.1002/cncr.32582 PMC6992519 31714597

[24] Forstner R. Early Detection of Ovarian Cancer. Eur Radiol (2020) 30:5370–3. doi: 10.1007/s00330-020-06937-z PMC7476911 32468105

[25] Chandra A Pius C Nabeel M Nair M Vishwanatha JK Ahmad S. Ovarian Cancer: Current Status and Strategies for Improving Therapeutic Outcomes. Cancer Med (2019) 8:7018–31. doi: 10.1002/cam4.2560 PMC6853829 31560828

[26] Chicago Consensus Working G. The Chicago Consensus on Peritoneal Surface Malignancies: Management of Ovarian Neoplasms. Cancer (2020) 126:2553–60. doi: 10.1002/cncr.32867 32282068

[27] Dilley J Burnell M Gentry-Maharaj A Ryan A Neophytou C Apostolidou S. Ovarian Cancer Symptoms, Routes to Diagnosis and Survival - Population Cohort Study in the ‘No Screen’ Arm of the UK Collaborative Trial of Ovarian Cancer Screening (UKCTOCS). Gynecol Oncol (2020) 158:316–22. doi: 10.1016/j.ygyno.2020.05.002 PMC7453382 32561125

[28] Perales-Puchalt A Wojtak K Duperret EK Yang X Slager AM Yan J. Engineered DNA Vaccination Against Follicle-Stimulating Hormone Receptor Delays Ovarian Cancer Progression in Animal Models. Mol Ther (2019) 27:314–25. doi: 10.1016/j.ymthe.2018.11.014 PMC6369450 30554854

[29] Garcia Prado J Gonzalez Hernando C Varillas Delgado D Saiz Martinez R Bhosale P Blazquez Sanchez J. Diffusion-Weighted Magnetic Resonance Imaging in Peritoneal Carcinomatosis From Suspected Ovarian Cancer: Diagnostic Performance in Correlation With Surgical Findings. Eur J Radiol (2019) 121:108696. doi: 10.1016/j.ejrad.2019.108696 31683251

[30] Jonsdottir B Ripoll MA Bergman A Silins I Poromaa IS Ahlstrom H. Validation of (18)F-FDG PET/MRI and Diffusion-Weighted MRI for Estimating the Extent of Peritoneal Carcinomatosis in Ovarian and Endometrial Cancer -a Pilot Study. Cancer Imaging (2021) 21:34. doi: 10.1186/s40644-021-00399-2 33849649 PMC8042953

[31] Wang R Cai Y Lee IK Hu R Purkayastha S Pan I. Evaluation of a Convolutional Neural Network for Ovarian Tumor Differentiation Based on Magnetic Resonance Imaging. Eur Radiol (2021) 31:4960–71. doi: 10.1007/s00330-020-07266-x 33052463

[32] Rizzo S De Piano F Buscarino V Pagan E Bagnardi V Zanagnolo V. Pre-Operative Evaluation of Epithelial Ovarian Cancer Patients: Role of Whole Body Diffusion Weighted Imaging MR and CT Scans in the Selection of Patients Suitable for Primary Debulking Surgery. A Single-Centre Study. Eur J Radiol (2020) 123:108786. doi: 10.1016/j.ejrad.2019.108786 31862634

[33] Zhang K Zhang Y Fang X Fang M Shi B Dong J. Nomograms of Combining Apparent Diffusion Coefficient Value and Radiomics for Preoperative Risk Evaluation in Endometrial Carcinoma. Front Oncol (2021) 11:705456. doi: 10.3389/fonc.2021.705456 34386425 PMC8353445

